# Mycobacteriosis in a Pet Ferret (*Mustela putorius furo*) Caused by *Mycobacterium xenopi*: A Case Report on Neglected Risk of Zoonotic Transmission

**DOI:** 10.3390/pathogens13040328

**Published:** 2024-04-16

**Authors:** Željko Mihaljević, Irena Reil, Josipa Habuš, Zrinka Štritof, Šimun Naletilić, Gabrijela Jurkić Krsteska, Tajna Kovač, Maja Zdelar-Tuk, Sanja Duvnjak, Silvio Špičić

**Affiliations:** 1 Croatian Veterinary Institute, Savska Cesta 143, 10000 Zagreb, Croatia; miha@veinst.hr (Ž.M.); zdelar-tuk@veinst.hr (M.Z.-T.); marjanovic@veinst.hr (S.D.); spicic@veinst.hr (S.Š.); 2 Faculty of Veterinary Medicine, University of Zagreb, Heinzelova 55, 10000 Zagreb, Croatia; jhabus@vef.unizg.hr (J.H.); zstritof@vef.unizg.hr (Z.Š.); gjurkic@vef.hr (G.J.K.); 3 Veterinary Clinic Dr. Pezo, Ede Murtica 2, 10000 Zagreb, Croatia; tajna.kovac@gmail.com

**Keywords:** ferrets, *Mycobacterium xenopi*, non-tuberculous mycobacteria, one health, zoonotic disease

## Abstract

Ferrets are highly susceptible to a wide range of mycobacteria, mainly *M. bovis*, *M. avium*, and *M. triplex*. Therefore, ferrets pose a risk of transmission of mycobacteriosis, especially zoonotically relevant tuberculosis. The aim of this study was to describe the findings of *M. xenopi* mycobacteriosis in a pet ferret and emphasize its zoonotic potential. A pet ferret had a history of weight loss, apathy, hyporexia, and hair loss. Abdominal ultrasound revealed splenomegaly with two solid masses and cystic lesions of the liver. Fine-needle aspiration cytology revealed numerous acid-fast bacilli in epithelioid cells, thus leading to the suspicion of mycobacterial infection. Because of its poor general condition, the ferret was euthanized. Necropsy examination revealed generalized granulomatous lymphadenitis, pneumonia, myocarditis, splenitis, and hepatitis. Histologically, in all organs, there were multifocal to coalescing areas of inflammatory infiltration composed of epithelioid macrophages, a low number of lymphocytes, and plasma cells, without necrosis nor multinucleated giant cells. Ziehl–Neelsen staining detected the presence of numerous (multibacillary) acid-fast bacteria, which were PCR-typed as *M. xenopi*. This is the first study showing the antimicrobial susceptibility testing of *M. xenopi* in veterinary medicine, describing the resistance to doxycycline. Overall, our results could facilitate further diagnosis and provide guidelines for the treatment protocols for such infections.

## 1. Introduction

The genus *Mycobacterium* is divided into members of the *Mycobacterium tuberculosis* complex (MTBC) and non-tuberculous mycobacteria (NTM). Ferrets are considered highly susceptible to a wide range of mycobacteria, with the most commonly reported cases of infection involving species *Mycobacterium bovis*, *M. avium*, and *M. triplex* [1]. Other reported mycobacterial infection isolates in ferrets include *M. genavense*, *M. microti*, *M. celatum*, *M. abscessus*, *M. fortuitum*, *M. florentinum*, *M. interjectum*, *M. septicum*, *M. peregrinum,* and *M. xenopi*. [2,3,4,5,6]. Infections caused by *M. bovis* in some ferrets were described as systemic disease, while in others, only one or more lymph nodes were affected. However, infections caused by *M. avium* complex are mostly described as granulomatous enteritis and pneumonia [2]. So far, mycobacteriosis has been regularly described and is widespread in feral ferrets in New Zealand, while in other countries, only sporadic occurrences have been described [7]. These infections in ferrets most often occur through ingestion, although infection through inhalation is also possible [8]. The disease most often causes granulomatous formations in the digestive tract and associated lymph nodes, but the spleen, liver, and lungs can also be affected, depending on the route of infection entry [8,9,10,11]. The mycobacteria were isolated from the oral cavity and bronchoalveolar lavage but also from urine, mammary tissue, and faeces of infected animals, indicating the sources of mycobacterial excretion [6].

## 2. Materials and Methods

### 2.1. In Vivo Clinical Examination 

We conducted a clinical study of mycobacteriosis in a male ferret, with further information provided later in the text. After arriving at the clinic, the animal underwent haematological and biochemical tests and an ultrasound examination. A cytological examination of suspicious tissues was also performed, and the obtained material was stained with the May–Grünwald–Giemsa (MGG) and Ziehl–Neelsen (ZN) stains. Amplification of the DNA sequence containing the gene coding for the 65 kDa antigen common in all mycobacteria was performed using conventional PCR to detect members of the genus *Mycobacterium* in punctate [12,13]. 

### 2.2. Post-Mortem Examination

After humane euthanasia, necropsy and histopathological examination were performed. Representative samples of lung, spleen, liver, and mesenteric and tracheobronchial lymph nodes were submitted for bacteriological examination and inoculated onto standard growth medium (Löwenstein–Jensen medium supplemented with pyruvate, Löwenstein–Jensen medium supplemented with glycerine, and Stonebrink medium) specially used for the culture of *Mycobacterium* species, followed by incubation at 37 °C [14]. Grown colonies were stained using ZN to confirm the presence of acid-fast bacilli. Furthermore, amplification of the DNA sequence containing the gene coding 65 kDa antigen was used to identify the grown colonies as members of the genus *Mycobacterium*. Isolated mycobacteria were tested with GenoType^®^ Mycobacterium CM (Hain Lifescience, Nehren, Germany) for further identification [12,13,15].

The obtained isolate was subjected to antimicrobial susceptibility testing via broth microdilution method using the Sensititre ™ Myco SLOMYCO AST Plate commercial kit (Thermo Fisher Scientific, Waltham, MA, USA) according to the Clinical and Laboratory Standards Institute recommendations [16,17]. Thirteen antibiotics most often used in human medicine were tested: amikacin (AMI), ciprofloxacin (CIP), clarithromycin (CLA), doxycycline (DOX), ethambutol (EMB), ethionamide (ETH), isoniazid (INH), linezolid (LZD), moxifloxacin (MXF), rifabutin (RFB), rifampin (RIF), streptomycin (STR), and trimethoprim-sulfamethoxazole (SXT). Cation-adjusted Mueller–Hinton broth (Thermo Fisher Scientific) supplemented with 5% Middlebrook Oleic Albumin Dextrose Catalase Growth Supplement (Sigma-Aldrich, St. Louis, MO, USA) was used for the preparation of bacterial suspension. Then microplate was incubated at 36 ± 1 °C for 14 days, followed by the classification of isolates as susceptible, intermediate susceptible, or resistant for all tested antibiotics except for EMB, ETH, and STR (no interpretation criteria have been established so far) [17].

## 3. Case Description

### 3.1. Patient

A 4-year-old hormonally castrated male ferret with a 3-month history of progressive weight loss, apathy, hyporexia, and hair loss was presented. The animal had been kept together with a female ferret for four years in the owner’s house (in a cage with temporary access to free range in the house) and was fed with commercial feed. The second female ferret had a diagnosis of lymphoma and was humanely euthanized due to poor health conditions and poor prognosis. Both ferrets were obtained from the same breeder from the ferret farm as cubs and were regularly vaccinated against distemper. Besides the ferrets, a 3-year-old female mixed-breed dog was present in the household.

### 3.2. Diagnostics

**Figure 1:**
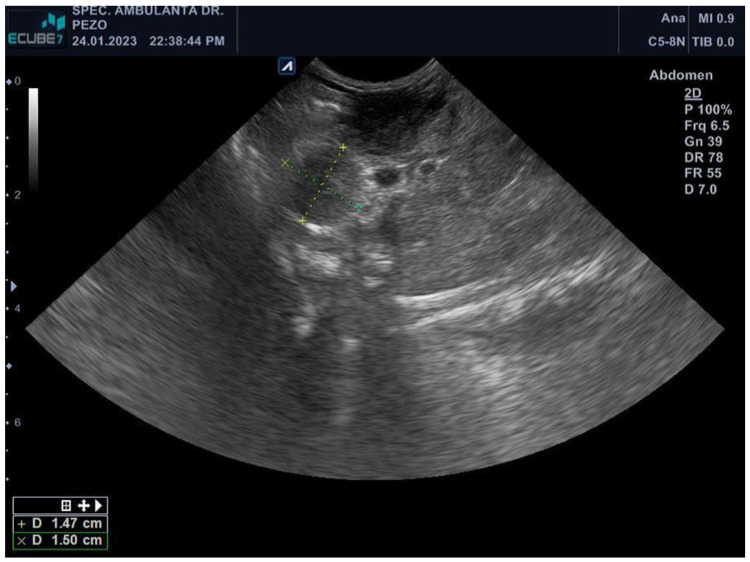
Abdominal ultrasound (*E*-*Cube 7*, *Alpinion*, Microconvex–array ultrasound transducer *C5*-*8N*) revealing two hyperechoic round masses measuring 1.56 × 1.31 cm and 1.47 × 1.5 cm in the pancreaticoduodenal area.

The ferret was depressed, hypothermic (rectal temperature of 36.2 °C), had a body condition score of 2 out of 5, and bilateral serous epiphora. A complete blood count revealed elevated values of white blood cells (WBCs) (17.8 10^9^/L, RI 2.5–5.5 10^9^/L), lymphocytes (LYM) (4.5 10^9^/L, RI 0.3–1.3 10^9^/L), granulocytes (GRA) (12 10^9^/L, RI 0.4–2.0 10^9^/L), monocytes (MON) (1.3 10^9^/L, RI 0.0–0.2 10^9^/L), and red cell distribution width (RDW) (17.10%, RI 14–17%), while decreased values of haemoglobin (HGB) (147 g/L, RI 150–180 g/L), mean corpuscular haemoglobin (MCH) (13.7 pg, RI 15–20 pg), and mean corpuscular haemoglobin concentration (MCHC) (290 g/L, RI 300–340 g/L) [18,19]. Other biochemical parameters showed elevated values of gamma-glutamyl transferase (GGT) (0.55 ukat/L, RI 0.00–0.03 ukat/L), cholesterol (CHOL) (8.70 mmol/L, RI 1.29–5.96 mmol/L), lipase (LIPA) (1.17 ukat/L, RI 0.00–0.53 ukat/L), and blood urea nitrogen (BUN) (15.30 mmol/L, RI 5.40–13.20 mmol/L), while decreased values of creatinine (CREA) (27.00 umol/L, RI 62.00–186.00 umol/L) and amylase (AMY) (1.53 ukat/L, RI 8.43–23.34 ukat/L). Palpation revealed enlarged popliteal lymph nodes. Abdominal ultrasound examination showed two hyperechoic round masses of 1.56 × 1.31 cm and 1.47 × 1.5 cm in the pancreaticoduodenal area (lymphadenomegaly) (Figure 1), cystic lesions of the liver, and splenomegaly. Fine-needle aspiration of the pancreaticoduodenal enlarged lymph nodes was performed, and smears were stained with the routine MGG stain. Cytologic examination revealed numerous epithelioid macrophages admixed with small lymphocytes, containing intracytoplasmic negative-staining rods measuring 2 × 0.5 μm. Staining with the ZN stain showed numerous acid-fast bacilli within the macrophages. Members of the genus *Mycobacterium* were identified in a punctate specimen by amplifying the 65 kDA antigen-specific DNA sequence. The animal was treated with amoxicillin-clavulanate (22 mg/kg every 12 h for 10 days) and, after the *Mycobacterium* determination, with enrofloxacin (10 mg/kg/day). The ferret became very weak and ill and had hypothermia and pale mucus membranes. He lost his appetite and rejected force-feeding. The ferret was humanely euthanized due to the poor prognosis and progressive clinical deterioration despite therapy.

### 3.3. Necropsy Findings

**Figure 2:**
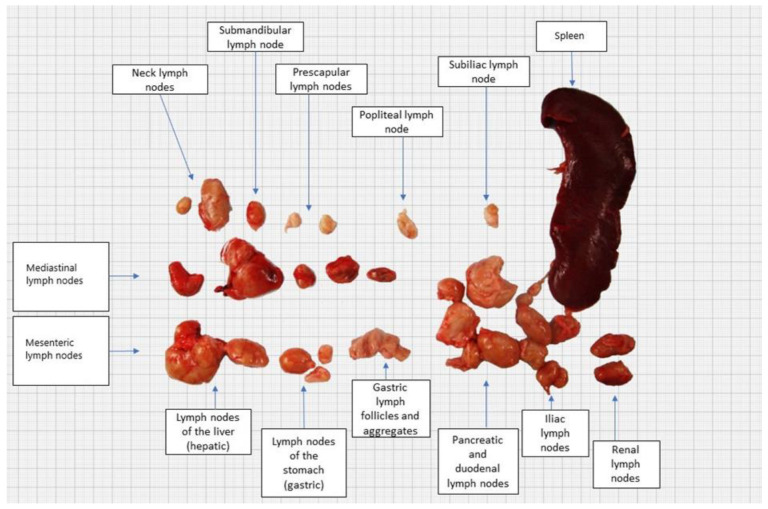
Gross appearance of enlarged lymph nodes and spleen.

**Figure 3:**
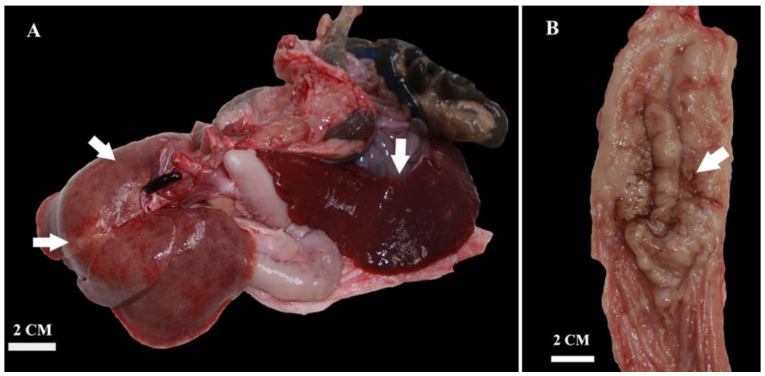
Gross appearance of the liver showing multifocal to coalescing gray to yellow areas and severely enlarged spleen (splenomegaly) (**A**). Diffuse thickening and multifocal ulcerations of gastric mucosa (**B**).

**Figure 4:**
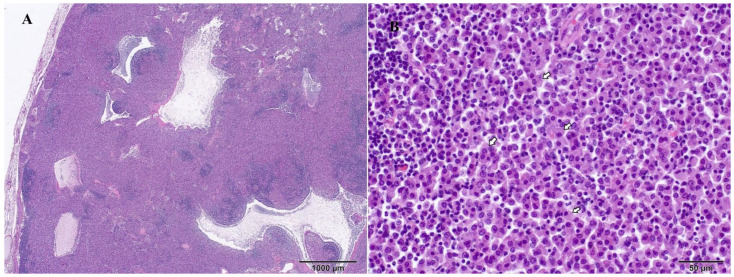
Mesenteric lymph node, ferret, and H&E stains. Large multifocal to coalescing area of epithelioid macrophage infiltration that disrupts lymph node architectures (**A**). Higher magnification shows epithelioid macrophages with large, bright cytoplasm that infiltrate the lymph node (**B**).

At post-mortem examination, all subcutaneous lymph nodes were moderately enlarged. The mesenteric and pancreaticoduodenal lymph nodes were markedly enlarged, whereas the mediastinal lymph node was moderately enlarged (Figure 2 and Figure 3). The lungs were voluminous, edematous, full of blood, and diffusely dark red color (edema and congestion, likely due to the euthanasia method), with prominent lobules. In cross-section, light yellow to pink areas were observed around the bronchus and bronchioles. The liver was slightly enlarged, with a pink to light brown color and numerous irregular multifocal to coalescing gray to yellow areas extending through the liver parenchyma. The spleen was markedly enlarged, dark red to dark brown in color, with a smooth capsule and multifocal light-yellow areas in the parenchyma measuring 0.3–0.5 cm in diameter. On the cut section, there was prominent white pulp hyperplasia. The gastric and small intestinal mucosa were moderately thickened with multifocal ulcerations (Figure 4).

### 3.4. Histopathological Findings

**Figure 5:**
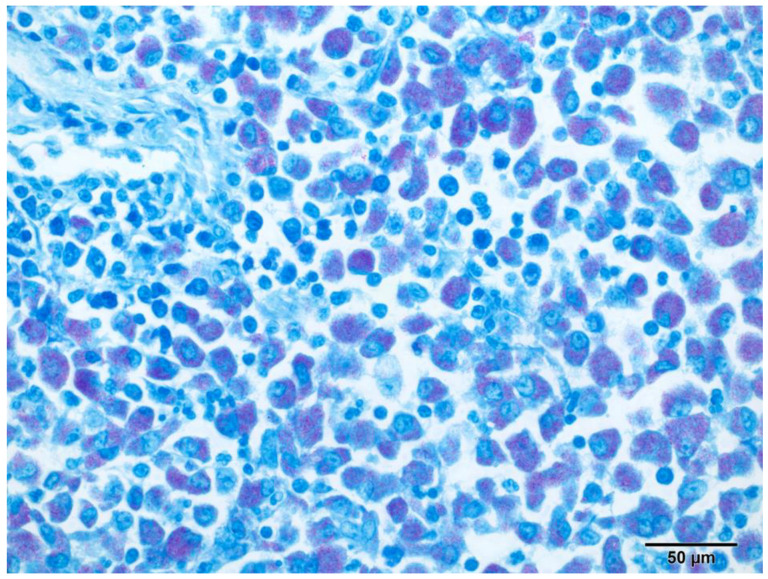
Pancreaticoduodenal lymph node, ferret. Abundant (multibacillary) slender rod-shaped intracytoplasmic acid-fast *M. xenopi* within infiltrating macrophages. Ziehl–Neelsen, original magnification 200×.

Representative samples from the mesenteric and mediastinal lymph nodes, stomach, liver, spleen, pancreas, intestine, heart, lung, kidney, and brain were fixed in 10% neutral-buffered formalin and routinely processed for histologic examination. Microscopically, samples from all organs revealed the presence of multifocal to coalescing poorly circumscribed areas of granulomatous inflammation containing numerous epithelioid macrophages (Figure 4) filled with myriad intracytoplasmic ZN-positive rod-shaped bacteria (Figure 5). There were no visible areas of necrosis among the granulomatous inflammation or multinucleated cells. Disseminated areas of granulomatous inflammation were observed around blood vessels in the lung, measuring 50 to 150 µm. The spleen also had marked extramedullary hematopoiesis (this is a common, likely incidental finding in ferrets).

### 3.5. Bacterial Examination and Molecular Identification

Grown colonies appeared on Löwenstein–Jensen medium supplemented with pyruvate on the 38th day of incubation, and the presence of acid-fast bacilli was determined using ZN staining. Amplification of the DNA sequence containing the gene coding for the 65 kDa antigen identified the grown colonies as a member of the genus *Mycobacterium*. The Geno Type^®^ Mycobacterium CM hybridization test (Hain Lifescience, Nehren, Germany) revealed that the isolate belonged to *M. xenopi*, which was confirmed in all tested tissues.

Regarding antimicrobial susceptibility testing, the *M. xenopi* isolate was susceptible to all tested antibiotics except for DOX, where it proved to be resistant. For antibiotics EMB, ETH, and STR, we recorded only minimum inhibitory concentration (MIC) values because no interpretation criteria have been established so far (Table 1).

Representative samples from the second female ferret were also subjected to a bacteriological examination, which ended with a negative result.

## 4. Discussion

Ferrets are particularly susceptible to a wide range of mycobacteria, with the most common ones being *M. bovis* and *M. avium* [7], followed by *M. genavense*, *M. celatum*, and *M. microti* [5,6,20]. Our case provides a clinical, ultrasonographic, cytologic, macroscopic, microscopic, and molecular description of *M. xenopi* in a ferret showing lesions that were more severe in the mesenteric and pancreaticoduodenal lymph nodes, stomach, intestines, spleen, and liver than the lung, suggesting a primary oral route of exposure, as most often diagnosed in ferrets with other mycobacteriosis [1,2,3,6,8,9,11]. However, the previous two publications on *M. xenopi* infection in ferrets describe more pronounced pathological changes in the lungs, which differs from our case [3,21]. It is also interesting to note that only the male ferret suffered from mycobacteriosis with a rapidly developing clinical picture, while the female was not infected, nor were mycobacteria isolated from her sample, even though she had been diagnosed with lymphoma. The animals were kept strictly indoors, and the source of infection has not been determined. Because of this, many issues from the epidemiological side remain unclear, as well as the fact about possible, so far undiscovered predispositions that led to the mycobacteria infection. The question of individual susceptibility to this infection remains unanswered, which indicates the need for further research and publication of such clinical cases.

In human medicine, *M. xenopi* is associated with the highest mortality among pulmonary nontuberculous mycobacterial (NTM) infections and is the second most commonly isolated NTM species responsible for pulmonary infection, behind *M. avium* complex [22]. A predisposing factor is immunosuppression, either local (lung disease) or systemic (lymphoproliferative malignancy, immunosuppressive therapy, or HIV infection) [3,23]. The preferred treatment regimen for *M. xenopi* infection in humans includes clarithromycin or azithromycin, ethambutol, and rifampin [24]. The isolate in our study did not show resistance to the mentioned antibiotics, but we should be aware that the treatment protocol for such infection is long-term with a very low success rate; in some studies, only 8.8% [25]. *M. xenopi* is an emerging opportunistic pathogen and should be considered as a potential zoonotic agent [2,3]. Although we have no confirmed disease in the owner of the ferret, we should be aware that the onset of symptoms is insidious, and the infection may progress slowly or increase and decrease over the months or years [26]. The rates of NTM lung diseases, of which *M. xenopi* is one of the most common causes, increase with human age and chronic diseases as well as menopause in women [27,28]. In this study, the organs of the ferret’s digestive tract were most affected, which indicates their excretion in faeces and the consequent contamination of the living space, thus exposing the owners and others who come into contact since infection occurs mainly through ingestion, inhalation, or direct inoculation.

Owners should be warned of the potential zoonotic risks if an infection with *M. xenopi* is suspected or detected in their pets. The diagnostic challenge for veterinary practice is the similarity of clinical signs and necropsy findings with lymphoma and systemic coronavirus-associated diseases. In our case, cytology and especially histopathologic evaluation were important tools to confirm the diagnosis of mycobacterial infection and to exclude the most important clinical differential diagnoses. Fine-needle aspiration of a lymph node or granuloma, stained with the ZN stain, also allows rapid, minimally invasive detection of acid-fast bacilli in macrophages and should be considered as a recommended procedure in the diagnostic process. PCR and molecular species typing via 16S rDNA sequencing are essential for a definitive etiologic diagnosis.

In conclusion, our results confirm the first detection of *M. xenopi* infection in a pet ferret in Croatia and present for the first time antimicrobial susceptibility testing for *M. xenopi* in veterinary medicine in general. Our study emphasizes the crucial importance of early detection of such infection and provides guidelines for rapid diagnosis. We should realize that this disease is a potential zoonotic threat to the most vulnerable groups and should not be ignored.

## Figures and Tables

**Table 1:** Breakpoints used for *M. xenopi* drug susceptibility testing and minimum inhibitory concentration (MIC) values of all drugs included in the panel.

Antimicrobial	MIC (µg/mL) Criteria			MIC Results	Interpretation
	S	I	R		
Amikacin	≤16	32	≥64	4	S
Ciprofloxacin	≤1	2	≥4	1	S
Clarithromycin	≤8	16	≥32	≤0.6	S
Doxycycline	≤1	2–4	≥8	8	R
Ethambutol	No interpretations available			8	/
Ethionamide	No interpretations available			5	/
Isoniazid	No interpretations available			2	S
Linezolid	≤8	16	≥32	4	S
Moxifloxacin	≤1	2	≥4	0.25	S
Rifabutin	≤2	-	≥4	≤0.25	S
Rifampin	≤1	-	≥2	1	S
Streptomicin	No interpretations available			8	/
Trimethoprim/sulfamethoxazole	≤2/38	-	≥4/76	0.25/4.75	S

S—susceptible; I—intermediate susceptible; R—resistant; MIC—minimum inhibitory concentration.

## Data Availability

The datasets generated and/or analyzed during the current study are not publicly available due to different institutional practices of data storage and access but are available from the corresponding author upon reasonable request.
